# OTUB1-SLC7A11 Axis Mediates 4-Octyl Itaconate Protection Against Acetaminophen-Induced Ferroptotic Liver Injury

**DOI:** 10.3390/antiox14060698

**Published:** 2025-06-09

**Authors:** Ziyun Hu, Yuxin Li, Di Xu, Huihui Meng, Wenya Liu, Qian Xu, Benxing Yao, Junsong Wang

**Affiliations:** Center for Molecular Metabolism, Nanjing University of Science and Technology, 200 Xiao Ling Wei Street, Nanjing 210094, China; hzy@njust.edu.cn (Z.H.); liyuxin@njust.edu.cn (Y.L.); xudi@njust.edu.cn (D.X.); menghuihui2014@gmail.com (H.M.); wenyaliu2015@163.com (W.L.); zhiqidangran@gmail.com (Q.X.); yaobenxing@njust.edu.cn (B.Y.)

**Keywords:** ferroptosis regulation, OTUB1-SLC7A11 axis, acetaminophen-induced hepatotoxicity, 4-octyl itaconate, iron homeostasis

## Abstract

Ferroptosis, an iron-dependent form of regulated cell death characterized by lipid peroxidation, plays a crucial role in acetaminophen (APAP)-induced hepatotoxicity. While 4-octyl itaconate (4-OI) demonstrates protective effects against APAP toxicity, its molecular mechanisms remain to be fully elucidated. Through an innovative integration of untargeted metabolomics and pathway analysis, we unveil a novel dual mechanism by which 4-OI prevents APAP-induced ferroptosis. We discovered that 4-OI stabilizes SLC7A11 through OTUB1-mediated deubiquitination, thereby restoring cystine import and glutathione (GSH) synthesis. In addition, 4-OI activates the Nrf2 pathway, orchestrating a comprehensive antioxidant response by upregulating the key proteins involved in both glutathione metabolism and iron homeostasis, including GPX4, FTH1, FTL1, and FPN1. This coordinated action effectively prevents the accumulation of toxic iron and lipid peroxides. Our findings not only elucidate the protective mechanisms of 4-OI but also establish it as a promising therapeutic candidate for ferroptosis-related diseases through its unique ability to simultaneously modulate the SLC7A11-GPX4 antioxidant axis and iron homeostasis.

## 1. Introduction

Ferroptosis is a novel form of iron-dependent cell death characterized by lipid peroxidation [1]. It is triggered by an inhibition of system X_C_^−^-mediated cystine uptake, which can impair glutathione (GSH) biosynthesis, as well as inhibit the GSH-dependent antioxidant enzyme GPX4 [2], leading to an iron-dependent accumulation of lipid peroxides and cell membrane damage. The iron-dependent nature of ferroptosis is tightly regulated by various proteins involved in iron metabolism, including transferrin for iron transport [3], transferrin receptor 1 (TFR1) for uptake [4], ferroportin 1 (FPN1) for cellular iron export [5], and ferritin for iron storage [6]. The dysregulation of iron homeostasis can result in iron overload, which catalyzes lipid peroxidation through the Fenton reaction and subsequently triggers ferroptosis [7].

Among the various conditions associated with ferroptosis, drug-induced liver injury has emerged as a serious concern. Acetaminophen (APAP), one of the most widely used over-the-counter analgesics for pain and fever relief, can trigger ferroptotic cell death in hepatocytes under overdose conditions. While APAP is safely metabolized through conjugation pathways at therapeutic doses, its overdose overwhelms these detoxification mechanisms, leading to the production of reactive metabolites such as N-acetyl-p-benzoquinone imine (NAPQI). This excessive NAPQI production results in GSH depletion and subsequent ferroptotic cell death [8,9,10,11,12].

In our previous research, we identified itaconate, an endogenous metabolite produced through the decarboxylation of cis-aconitate in the tricarboxylic acid (TCA) cycle by immunoresponsive gene 1 (IRG1), as a potential therapeutic agent [13]. Specifically, 4-octyl itaconate (4-OI), a cell-permeable derivative of itaconate, demonstrated promising effects in attenuating APAP-induced liver injury [14]. However, the precise molecular mechanisms underlying this protective effect, particularly in relation to ferroptosis, remain to be fully elucidated.

This study employs an integrated metabolomics and pathway analysis strategy to elucidate the hepatoprotective mechanisms of 4-OI in APAP-triggered ferroptotic liver damage. By examining the alterations in endogenous metabolite levels induced by APAP and their modulation by 4-OI treatment, we seek to uncover how 4-OI exerts its protective effects at the metabolic level and explore the potential involvement of ferroptosis. This research provides novel insights into the anti-ferroptotic mechanisms of 4-OI and its promising therapeutic potential for the treatment of ferroptosis-related liver diseases.

## 2. Materials and Methods

### 2.1. Reagents and Antibodies

The ferroptosis inhibitor Ferrostatin-1 (Fer-1), 4-OI (CAS: 3133-16-2, Figure 1A), the SLC7A11 inhibitor HG106, and the Nrf2 inhibitor ML385 were provided by MCE (Monmouth Junction, NJ, USA). APAP was obtained from Aladdin Chemical Reagent Co., Ltd. (Shanghai, China). Erastin, a well-established and widely used inducer of ferroptosis, was obtained from McLean (Shanghai, China). Fetal bovine serum (FBS) and RPMI-1640 were provided by Gibco (Carlsbad, CA, USA). Penicillin–streptomycin was obtained from HyClone (Victoria, Australia). The MTT cell proliferation and cytotoxicity assay kit, GSH and GSSG assay kit, DAPI, and DCFH-DA were purchased from Beyotime Biotechnology (Shanghai, China). N-ethylmaleimide (NEM) was purchased from Sigma-Aldrich (Saint Louis, MO, USA). Primary antibodies for SLC7A11, OTUB1, CD44, ubiquitin, GPX4, Nrf2, transferrin, TFR1, FTH1, FTL1, FPN1, hepcidin, and GAPDH were obtained from the following sources: SLC7A11 and OTUB1 antibodies from Abcam (Cambridge, UK); hepcidin antibody from Affinity (Changzhou, China); CD44, ubiquitin, GPX4, Nrf2, transferrin, TFR1, FTH1, FTL1, FPN1, and GAPDH antibodies from Proteintech (Wuhan, China). The secondary antibodies anti-mouse and anti-rabbit were acquired from Proteintech (Wuhan, China). The BCA Protein Quantification Kit was obtained from Yeasen Biotechnology (Shanghai Co., Ltd., Shanghai, China), while the Tissue Iron Assay Kit and Serum Iron Assay Kit were purchased from Nanjing Jiancheng Bioengineering Institute (Nanjing, China).

### 2.2. Animals and Treatments

Male C57BL/6J mice, weighing approximately 20–25 g and aged 6–8 weeks, were obtained from Qinglongshan Laboratory Animal Company (Nanjing, China). The animals were housed in a controlled environment, with the temperature maintained at 21–23 °C and humidity levels between 40–55%. After one week of acclimatization, mice were randomly assigned to seven experimental groups (*n* = 7 per group). Prior to the experiments, mice were subjected to overnight fasting but had unrestricted access to water. To establish experimental liver injury models, mice were subjected to one of two treatments: a single intraperitoneal (i.p.) injection of acetaminophen (APAP, 400 mg/kg) or four consecutive doses of erastin (50 mg/kg, i.p.) administered at 12 h intervals. In the pretreatment protocol, 4-OI (50 mg/kg, i.p.) was given 2 h before the initial erastin or APAP administration. As a positive control for ferroptosis inhibition, Fer-1 (5 mg/kg, i.p.) was administered 1 h prior to either treatment. Liver samples were collected 6 h after either the APAP injection or the final erastin dose. Following anesthesia, the livers were carefully excised, washed with ice-cold saline, and dried before weighing. The tissue samples were then either immediately snap-frozen in liquid nitrogen or preserved in 10% neutral-buffered formalin for subsequent analyses.

### 2.3. Cell Culture

Human hepatic cell line L02 and human hepatoma cell line HepG2 were obtained from the Shanghai Institute of Biochemistry and Cell Biology, Chinese Academy of Sciences. The cells were cultured in RPMI-1640 medium supplemented with 10% FBS, 100 units/mL penicillin, and 100 μg/mL streptomycin at 37 °C in a humidified atmosphere with 5% CO_2_.

### 2.4. Cell Viability Assay

L02 and HepG2 cells were seeded into 96-well plates and cultured for 24 h at 37 °C in a 5% CO_2_ incubator to allow cell attachment. The cells were then pretreated with 4-OI at concentrations of 25 and 50 μM for 12 h. Subsequently, APAP was added at a concentration of 10 mM for L02 cells or 20 mM for HepG2 cells, and the incubation was continued for an additional 24 h. Cell viability was assessed using the MTT assay. Briefly, MTT solution (5 mg/mL in PBS) was added to each well at a volume of 20 μL, followed by the incubation of the cells for an additional 4 h at 37 °C. Then, the culture medium containing the MTT solution was removed, and 150 μL of DMSO were added to solubilize the insoluble formazan crystals formed in each well. The absorbances of the resulting solutions were measured at a wavelength of 570 nm using a microplate reader to quantify cell viability.

### 2.5. Metabolomics Profiling

#### 2.5.1. Metabolites Extraction

Liver tissues were homogenized in 1 mL of an extraction solution containing methanol, acetonitrile, and water in a 2:2:1 ratio. L02 cells were subjected to three freeze–thaw cycles using 1 mL of the same methanol–acetonitrile–water extraction solution. The resulting mixtures were then stored at −20 °C for 1 h to precipitate the proteins. After centrifugation at 16,000× *g* for 15 min at 4 °C, the supernatants were collected and dried using a vacuum concentrator. The dried samples were reconstituted by adding 100 μL of a 1:1 acetonitrile–water solution and vortex-mixed again for 30 s. Subsequently, the reconstituted samples were sonicated for 10 min at 4 °C, followed by centrifugation at 16,000× *g* for 15 min at 4 °C. The resulting supernatants (60 μL) were then subjected to UHPLC-QTOF-MS analysis.

#### 2.5.2. UHPLC-QTOF-MS Analysis

Metabolite profiling was conducted using an LC system coupled to a TripleTOF 5600^+^ mass spectrometer (AB Sciex, Framingham, MA, USA). Chromatographic separation was performed on an Atlantis^TM^ Premier BEH C18 AX column (2.1 × 100 mm, 1.7 μm; Waters, Milford, MA, USA) maintained at 40 °C. The mobile phase consisted of (A) 0.1% formic acid in water and (B) acetonitrile, with the following gradient: 0 min, 1% B; 1 min, 1% B; 10 min, 99% B; 13 min, 99% B; 14 min, 1% B; and 17 min, 1% B at 0.4 mL/min flow rate (injection volume: 10 μL). The information-dependent acquisition (IDA) mode was utilized to obtain the MS/MS spectra during the LC/MS runs on the TripleTOF 5600^+^. The Analyst software (version 2.0) evaluated full-scan MS data in real-time and triggered MS/MS scans for the top 10 precursor ions at a collision energy (CE) of 35 ± 15 V. The electrospray ionization (ESI) source was operated in negative ion mode (−4000 V) and subsequently in positive ion mode (+5500 V) for the same sample batch, with the following shared parameters: source temperature at 550 °C, nebulizer gas at 55 psi, heater gas at 55 psi, and curtain gas at 35 psi.

#### 2.5.3. UHPLC-QTOF-MS Data Processing

Mass spectrometry raw data were first transformed into mzML format through ProteoWizard software (version 3.0. 4416). Subsequent data processing, including peak alignment, retention time adjustment, and peak area extraction, was conducted using R software (version 3.0.2). Metabolite identification was accomplished by matching the experimental MS/MS spectra against the reference spectra in the Human Metabolome Database (HMDB) with a secondary scoring system. The initial mass spectrometry screening maintained a stringent mass accuracy criterion of <10 ppm. For multivariate pattern recognition analysis, partial least squares–discriminant analysis (PLS-DA) was performed using the mixOmics package in the R software.

#### 2.5.4. Metabolic Set Enrichment Analysis (MSEA) and Pathway Analysis

MSEA was conducted to identify significantly enriched metabolic pathways associated with the differentially expressed metabolites. This analysis was facilitated through the MetaboAnalyst 5.0 platform, a comprehensive online tool designed for metabolomic data analysis.

Based on the Pathway Commons database (http://www.pathwaycommons.org/, accessed 12 March 2024), genes and proteins interacting with the differential metabolites were identified. These interacting genes and proteins were then subjected to pathway analysis using the R package CePa. Metabolites that exhibited post-treatment level changes falling between those of the control and APAP groups were categorized as efficacy-related, suggesting that the drug exerted a regulatory effect in restoring these metabolite levels toward normalcy. In contrast, metabolites whose levels remained consistently higher or lower in both the control and APAP groups after treatment were classified as drug bias-related metabolites, reflecting the inherent bias or specificity of the drug’s action.

### 2.6. ROS Detection

HepG2 cells were seeded in 6-well plates and pretreated with 25 μM 4-OI for 12 h. The cells were then exposed to 20 mM APAP for 24 h. To detect intracellular ROS, the HepG2 cells were stained with a 10 μM DCFH-DA solution in the dark at 37 °C for 20 min. Following incubation, the cells were washed with PBS to remove any residual DCFH-DA. The cells were then collected and immediately analyzed using an ACEA Novocyte flow cytometer (ACEA Biosciences Inc., San Diego, CA, USA). The acquired flow cytometry data were processed and analyzed using FlowJo software (version 10.6.2).

### 2.7. Quantitative Real-Time PCR (RT-qPCR)

Total RNA was isolated from liver tissues using Trizol reagent, followed by cDNA synthesis with a commercial kit. RT-qPCR was performed using KAPA SYBR FAST Master Mix (KAPA Biosystems, Wilmington, MA, USA) on a 7300 Real-Time PCR System, with GAPDH as the endogenous control. All primers (Tsingke Biotechnology, Nanjing, China) are listed in Supplementary Table S1.

### 2.8. Western Blot Analysis

Liver tissues or cells were homogenized in ice-cold RIPA buffer supplemented with protease inhibitors, and protein concentrations were quantified by a BCA assay. Protein samples were separated by 12% SDS-PAGE and subsequently electrotransferred to nitrocellulose membranes (Pall #66485). After blocking with 5% non-fat milk in TBST for 2 h at room temperature, membranes were incubated overnight at 4 °C with primary antibodies against: GPX4 (67763-1-Ig, 1:1000), SLC7A11 (ab175186, 1:1000), OTUB1 (ab270959, 1:1000), CD44 (60224-1-Ig, 1:1000), ubiquitin (10201-2-AP, 1:1000), Nrf2 (16396-1-AP, 1:1000), transferrin (17435-1-AP, 1:1000), TFR1 (10084-2-AP, 1:5000), FTH1 (11682-1-AP, 1:1000), FTL1 (10727-1-AP, 1:1000), FPN1 (26601-1-AP, 1:1000), hepcidin (DF6492, 1:500), and GAPDH (60004-1-Ig, 1:2000). Following primary antibody incubation, membranes were treated with horseradish peroxidase (HRP)-conjugated secondary antibodies (anti-rabbit SA00001-2 or anti-mouse SA00001-1, 1:5000) for 1 h at room temperature. Protein signals were detected using an enhanced chemiluminescence (ECL) substrate and imaged on a ChemiScope 6100 system (Clinx Science Instruments, Shanghai, China). Band intensities were quantified by densitometry using ImageJ (version 1.52a, NIH, USA).

### 2.9. Immunofluorescence Staining

L02 cells and liver tissue sections were fixed in 4% paraformaldehyde for 30 min at room temperature, followed by three 5-min washes with PBS to ensure complete fixative removal. The cells and tissues were then permeabilized with 0.1% Triton X-100 for 10 min to improve antibody penetration. Non-specific binding sites were blocked by incubating the samples with 4% bovine serum albumin (BSA) in 0.1% Triton X-100 for 40 min at room temperature. The samples were then incubated overnight at 4 °C with primary antibodies. On the following day, the samples were exposed to either fluorescein isothiocyanate (FITC)- or cyanine 3 (Cy3)-conjugated secondary antibodies in the dark for 1 h at room temperature. Nuclear staining was performed using DAPI for 10 min in the dark. Finally, the labeled samples were visualized and imaged using a fluorescence microscope.

### 2.10. Co-Immunoprecipitation (Co-IP) and Ubiquitination Assays

For Co-IP assays, the liver tissues were homogenized in RIPA lysis buffer supplemented with protease and phosphatase inhibitors, followed by incubation on ice for 30 min. The homogenates were then centrifuged at 16,000× *g* for 30 min at 4 °C. An aliquot equaling 5% of the resulting supernatant was collected and utilized as an input control for the subsequent immunoblot analyses. The remaining clarified lysates were incubated with antibodies targeting the proteins of interest and rotated at 4 °C for 30 min. Next, 50 μL of Protein A/G agarose beads (Santa Cruz Biotechnology, Dallas, TX, USA, sc-2003) were added to the mixtures, which were then incubated overnight at 4 °C to precipitate antibody–antigen complexes. The bead-bound immune complexes were stringently washed four times with lysis buffer and isolated by centrifugation. The complexes were subsequently dissociated from the beads by boiling in SDS-PAGE sample buffer, followed by a Western blot analysis. For the ubiquitination assay, 20 mM NEM were added to the RIPA lysis buffer.

### 2.11. OTUB1 Knockdown

Small interfering RNAs (siRNAs) targeting OTUB1 (siOTUB1) and a negative control siRNA (siNC) were obtained from Sangon Biotech (Shanghai, China). The siOTUB1 sequences were sense primer 5′-CAAGGAGUAUGCUGAAGAUTT-3′; antisense primer 5′-AUCUUCAGCAUACUCCUUGTT-3′. The siNC sequences were sense primer 5′-UUCUCCGAACGUGUCACGUTT-3′; antisense primer 5′-ACGUGACACGUUCGGAGAATT-3′. L02 cells were transfected with 50 nM siNC or siOTUB1 using ExFect Transfection Reagent (Vazyme, Cat#T101-02, Nanjing, China) for 48 h. Subsequently, the cells were harvested, and the knockdown efficiency was validated through RT-qPCR and Western blotting.

### 2.12. Hematoxylin–Eosin (H&E) Staining

For histological examination, paraffin-embedded liver tissue specimens underwent a systematic preparation process. The sections were first deparaffinized in xylene, then progressively rehydrated through a descending gradient of ethanol solutions. The rehydrated sections were stained with hematoxylin for 5 min, followed by differentiation in a 0.1% hydrochloric acid–alcohol solution. After counterstaining with eosin for 2 min, the sections were dehydrated through an ascending ethanol gradient. The processed sections were then cleared in xylene and permanently mounted on glass slides using neutral mounting medium. Histopathological evaluation and imaging were performed using a light microscope.

### 2.13. Immunohistochemistry (IHC)

Liver tissues were fixed in 4% paraformaldehyde and processed for paraffin embedding. Sections of 4 μm thickness were cut from the paraffin-embedded tissue blocks. Following deparaffinization and rehydration, the liver sections underwent antigen retrieval by heating in sodium citrate buffer (pH 6.0) for 20 min, followed by incubation with 3% H_2_O_2_ solution for 15 min to quench the endogenous peroxidase activity. The sections were then blocked with 10% goat serum for 1 h at room temperature. Subsequently, the liver sections were incubated overnight at 4 °C with primary antibodies. Following three PBS washes, the sections were incubated with HRP-conjugated secondary antibodies for 1 h at room temperature. The immunoreactive proteins were visualized by staining the sections with the chromogenic substrate 3,3′-diaminobenzidine (DAB), followed by counterstaining with hematoxylin. Finally, the stained sections were examined and imaged using a light microscope.

### 2.14. Measurement of Alanine Aminotransferase (ALT) and Aspartate Aminotransferase (AST) Levels

The blood samples were centrifuged (3000× *g*, 10 min, 25 °C) to obtain serum. The separated serum was analyzed using an automated biochemical analyzer to measure the activities of two hepatic injury markers: ALT and AST.

### 2.15. Measurement of Hepatic Iron Levels

The iron content in liver tissue samples or cells was quantified using a commercial iron assay kit, following the manufacturer’s protocols. Briefly, the liver tissues were homogenized in ice-cold extraction buffer and centrifuged at 600× *g* for 10 min at 4 °C to obtain the supernatants. The supernatants were then mixed with the supplied assay buffer and re-centrifuged at 1200× *g* for 10 min at 4 °C. Subsequently, the absorbance of the resulting solutions was measured at 520 nm using a microplate reader. The protein concentrations in the samples were determined using a BCA Protein Quantification Kit in accordance with the manufacturer’s instructions.

### 2.16. GSH Detection

GSH levels were quantified using a GSH and GSSG assay kit following the manufacturer’s instructions. L02 and HepG2 cells were treated with 25 μM 4-OI for 12 h, followed by the addition of HG106 (5 μM) for 12 h. The harvested cells or liver tissues were homogenized after adding 70 µL of protein-removal reagent. The homogenates were then centrifuged at 10,000× *g* for 10 min at 4 °C, and the resulting supernatant was collected for total GSH measurement. Subsequently, a GSH-scavenging working solution was added to the supernatant to eliminate GSH. Finally, the samples were measured at a wavelength of 412 nm, and the GSH levels were calculated based on measurements of total GSH and oxidized GSH.

### 2.17. Measurement of SOD and MDA Levels

The liver tissues or the collected cells were resuspended in ice-cold saline and subjected to homogenization. The homogenate was centrifuged at 1000× *g* for 10 min at 4 °C. The supernatant was used to measure the SOD activity and MDA content according to the manufacturer’s instructions (Nanjing Jiancheng Bioengineering Institute, Nanjing, China).

### 2.18. Statistical Analysis

All quantitative data are expressed as the mean ± SD derived from a minimum of three biological replicates. Statistical analyses were conducted using either GraphPad Prism (version 8.0) or R statistical software (version 3.0.2). For comparisons involving two experimental groups, a two-tailed unpaired Student’s *t*-test was employed. Multiple group analyses were performed using a one-way ANOVA with a Tukey’s post hoc test, following a verification of normality and equal variance assumptions. When the parametric test assumptions were not satisfied, we applied either appropriate data transformations or non-parametric alternatives. Statistical significance was defined as *p* < 0.05 for all analyses.

## 3. Results

### 3.1. 4-OI Protects Against APAP-Induced Liver Injury by Modulating Inflammatory Response and Oxidative Stress

**Figure 1:**
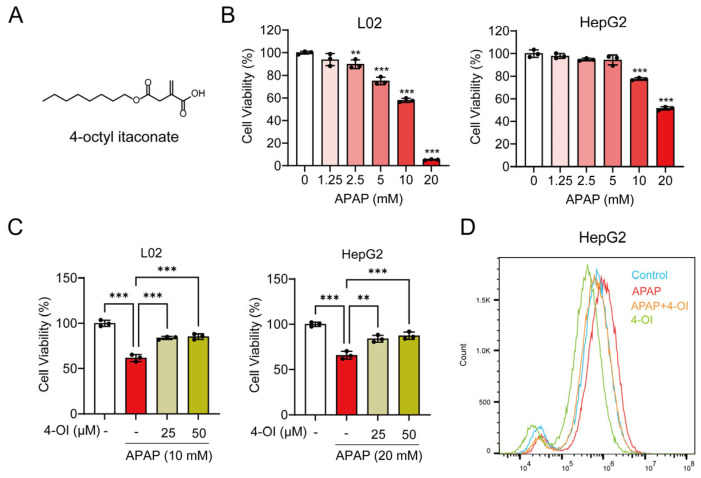
4-OI attenuates APAP-induced hepatotoxicity. (**A**) Chemical structure of 4-OI. (**B**) MTT assay showing viability of L02 and HepG2 cells treated with APAP (0, 1.25, 2.5, 5, 10, 20 mM) for 24 h (*n* = 3). ** *p* < 0.01, *** *p* < 0.001 compared with the untreated cells group. (**C**) MTT assay evaluated the viability of L02 and HepG2 cells that were pretreated with 25 or 50 μM 4-OI for 12 h before exposure to APAP (*n* = 3). (**D**) Intracellular ROS levels in HepG2 cells were detected by flow cytometry and analyzed using FlowJo software (version 10.6.2) (*n* = 3). ** *p* < 0.01, *** *p* < 0.001.

Our previous study demonstrated that 4-OI reduces APAP-induced liver injury, as evidenced by attenuated hepatocellular necrosis and decreased serum liver enzyme levels. We also established that 50 μM is a safe and non-cytotoxic concentration of 4-OI for L02 and HepG2 cells [14]. Building upon these findings, we sought to further investigate the protective mechanisms of 4-OI against APAP-induced liver injury. We discovered that 4-OI not only attenuates APAP-induced oxidative stress in the liver but also significantly modulates inflammatory responses. APAP treatment markedly induced the expression of the pro-inflammatory cytokines *TNF-α*, *IL-1β*, and *IL-6* in the mouse liver, while an administration of 4-OI effectively suppressed the APAP-induced upregulation of these cytokines (Figure S1A–C). Furthermore, we found that APAP exposure led to a substantial decrease in the GSH/GSSG ratio, which was significantly reversed by 4-OI pretreatment (Figure S1D). To establish an in vitro model of APAP-induced hepatocellular injury, we treated L02 and HepG2 cells with increasing concentrations of APAP (0, 1.25, 2.5, 5, 10, and 20 mM). A cell viability analysis revealed a dose-dependent cytotoxic effect of APAP, where L02 cell viability decreased to 57.94% of the control after 10 mM APAP treatment, while the HepG2 cell viability reduced to 51.44% of the control with 20 mM APAP (Figure 1B). Based on these results, we selected 10 mM APAP for L02 cells and 20 mM APAP for HepG2 cells to induce hepatocellular injury in subsequent experiments. We then investigated whether 4-OI could protect against APAP-induced cytotoxicity in hepatocytes. Pretreatment with 4-OI (25 and 50 μM) dose-dependently attenuated APAP-induced cell death in both L02 and HepG2 cells (Figure 1C). Additionally, a flow cytometry analysis revealed that 4-OI pretreatment significantly suppressed APAP-induced ROS generation in HepG2 cells (Figure 1D). Notably, treatment with 4-OI alone decreased ROS levels compared to the control group, suggesting that 4-OI possesses inherent antioxidant properties. Collectively, these results demonstrate that 4-OI protects against APAP-induced liver injury through both anti-inflammatory and antioxidant mechanisms, both in vivo and in vitro.

### 3.2. 4-OI Modulates Both Inflammatory and Antioxidant Metabolic Pathways in APAP-Induced Hepatotoxicity

**Figure 2:**
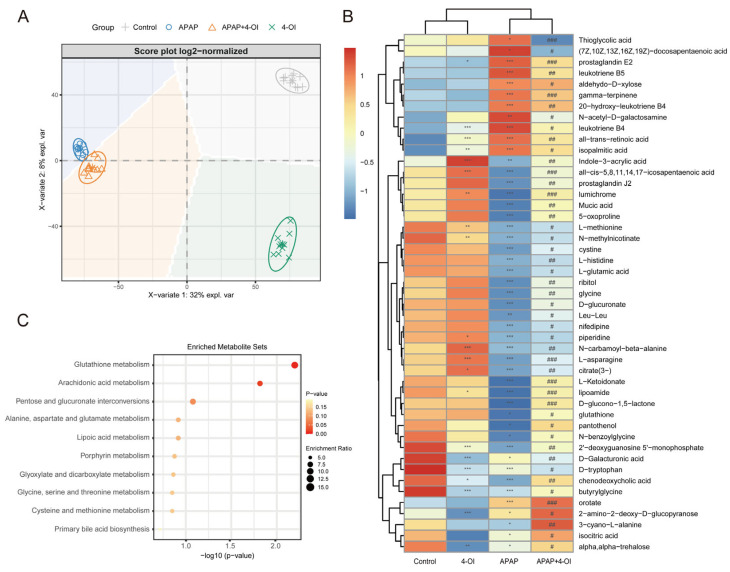
Metabolic landscape and pathway enrichment in APAP-exposed mice treated with 4-OI. (**A**) PLS-DA-based multivariate analysis of metabolic profiles revealed distinct clustering among experimental groups (*n* = 10 mice per group). The principal components (PCs) highlight key metabolic differences. PC1 (*X*-axis) represents drug bias, where metabolites with VIP > 1 show consistent deviations from both control and APAP groups, indicating unique molecular targets and mechanisms of action. PC2 (*Y*-axis) represents drug efficacy, where metabolites with VIP > 1 demonstrate normalization toward baseline levels, reflecting the therapeutic effect of 4-OI. (**B**) Hierarchically clustered heatmap displaying median-scaled metabolite levels, differentiating the control, APAP, APAP + 4-OI, and 4-OI groups (*n* = 7). Relative metabolite abundances are depicted in blue (low) and red (high). Statistical significance (adjusted *p*-values) was determined using the Benjamini–Hochberg method, with either a two-tailed Student’s *t*-test or Mann–Whitney test, as appropriate. (**C**) Top 10 pathways enriched over significantly changed metabolites. * *p* < 0.05, ** *p* < 0.01, *** *p* < 0.001 compared with the control group; # *p* < 0.05, ## *p* < 0.01, ### *p* < 0.001 compared with the APAP group.

To further elucidate the mechanisms underlying the hepatoprotective effects of 4-OI, we conducted a comprehensive metabolomic profiling using UHPLC-QTOF-MS to delineate the metabolic shifts in mouse livers following treatment with 4-OI and APAP. The PLS-DA score plot revealed distinct metabolic profiles among the control, APAP, APAP + 4-OI, and 4-OI groups, indicating significant divergence in their metabolic processes (Figure 2A). Our analysis focused on two key pharmacological properties: drug efficacy and drug bias. Drug efficacy was evaluated through metabolites that demonstrated 4-OI’s ability to normalize APAP-induced metabolic perturbations toward baseline levels. These efficacy-related metabolites exhibited post-treatment levels that were intermediate between the control and APAP groups (Figure 2A, PC2 direction, VIP > 1), reflecting 4-OI’s therapeutic effectiveness in restoring metabolic homeostasis. Drug bias was assessed through metabolites that revealed 4-OI’s unique molecular targets and mechanisms of action, independent of its therapeutic effects. These bias-related metabolites showed consistent deviations from both the control and APAP groups (Figure 2A, PC1 direction, VIP > 1), either displaying elevation or reduction, thereby highlighting the drug’s specific molecular targeting profile and distinct biological activity. Hierarchical clustering analysis further confirmed these distinct metabolite patterns across the treatment groups (Figure 2B). Notably, the APAP treatment substantially altered the levels of metabolites involved in multiple pathways. We observed marked increases in pro-inflammatory mediators, including (7*Z*,10*Z*,13*Z*,16*Z*,19*Z*)-docosapentaenoic acid, prostaglandin E2, leukotriene B5, 20-hydroxy-leukotriene B4, and leukotriene B4, all of which are key components of the arachidonic acid pathway associated with inflammatory responses. Importantly, 4-OI pretreatment effectively normalized these elevated inflammatory mediators. Additionally, APAP exposure significantly depleted the antioxidant-related metabolites, particularly those involved in glutathione metabolism, including L-methionine, L-cystine, and glutathione. Pretreatment with 4-OI restored these critical antioxidant metabolites to near-control levels, suggesting a robust antioxidant response. The heatmap also revealed alterations in amino acids and energy metabolism intermediates, indicating widespread metabolic perturbations induced by APAP that were largely reversed by 4-OI pretreatment. MSEA corroborated these findings, with glutathione metabolism and arachidonic acid metabolism emerging as the most significantly enriched pathways (Figure 2C). This analysis aligned well with the observed metabolite changes in the heatmap, particularly the restoration of glutathione-related metabolites and the normalization of inflammatory mediators by 4-OI. Collectively, these results demonstrate that 4-OI exerts its hepatoprotective effects through dual mechanisms: suppressing pro-inflammatory metabolite production while simultaneously preserving cellular antioxidant capacity by maintaining glutathione-related metabolite levels.

### 3.3. 4-OI Exerts Dual Properties of Iron Homeostasis Regulation During APAP-Induced Liver Injury

**Figure 3:**
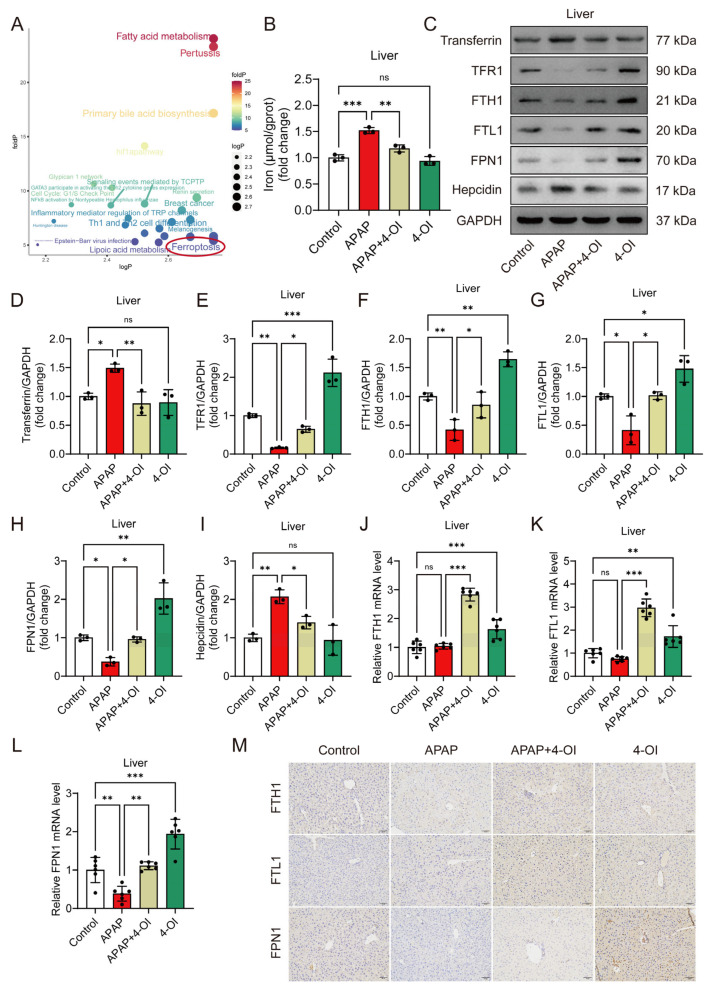
4-OI mitigates APAP-induced dysregulation of iron metabolism. (**A**) Pathways associated with genes/proteins interacting with differential metabolites were analyzed using the R package CePa. logP (*x*-axis): negative log of the enrichment *p*-value for drug bias-related metabolites. foldP (*y*-axis): ratio of enrichment *p*-values for efficacy-related vs. bias-related metabolites. The red circle highlights the ferroptosis pathway, which is identified as the most prominent among all analyzed pathways. (**B**) Hepatic iron content (*n* = 3). (**C**–**I**) Representative Western blot images and quantification of transferrin, TFR1, FTH1, FTL1, FPN1, and hepcidin protein levels in mouse liver tissues (*n* = 3). (**J**–**L**) A RT-qPCR assay determined the mRNA expression of *FTH1*, *FTL1*, and *FPN1* (*n* = 6). (**M**) Representative immunohistochemical staining images showing FTH1, FTL1, and FPN1 expression. Scale bar: 100 μm, *n* = 3. GAPDH was used as internal loading control. * *p* < 0.05, ** *p* < 0.01, *** *p* < 0.001; ns: not significant.

Differentially expressed metabolites were classified according to their response patterns. A pathway analysis of their corresponding upstream proteins identified ferroptosis as a significantly enriched pathway in both categories (Figure 3A), indicating that 4-OI influences iron homeostasis through both therapeutic and direct mechanisms. To validate these metabolomic findings, we developed an in vivo ferroptosis-induced liver injury model using erastin. Histological examination using H&E staining demonstrated that both 4-OI (50 mg/kg) and Fer-1 (5 mg/kg) mitigated erastin- or APAP-induced hepatic histopathological changes and cell death (Figure S2A). Liver function assessment revealed that both 4-OI and Fer-1 significantly reduced serum ALT and AST levels in mice treated with either erastin or APAP (Figure S2B,C). Moreover, both compounds restored the GSH/GSSG ratio and SOD activity while suppressing MDA accumulation in the livers of erastin- or APAP-treated mice (Figure S2D–F). Additionally, 4-OI restored the serum iron levels and reduced the elevated hepatic iron content caused by both erastin and APAP treatments (Figure S2G,H and Figure 3B). Further investigation of iron metabolism markers revealed two distinct mechanisms of 4-OI: efficacy-related and bias-related effects. The efficacy-related effects were evidenced by 4-OI’s ability to normalize APAP-induced changes, including the reversal of elevated transferrin and hepcidin levels, and the restoration of decreased TFR1, FTH1, FTL1, and FPN1 protein expression (Figure 3C–I). The bias-related effects were demonstrated by 4-OI’s actions independent of APAP treatment, where 4-OI alone significantly upregulated TFR1, FTH1, FTL1, and FPN1 expression compared to the control conditions (Figure 3C–I). These dual effects were further confirmed at the transcriptional level. Under APAP treatment, the mRNA expression of *FPN1* was significantly decreased compared to the control conditions, and 4-OI effectively reversed this decrease to near-control values. Moreover, treatment with 4-OI alone significantly enhanced the mRNA expression of *FTH1*, *FTL1*, and *FPN1* above the control levels (Figure 3J–L), demonstrating its independent regulatory effects on iron metabolism. Notably, we observed an interesting discrepancy between the protein and mRNA levels of FTH1 and FTL1 in the APAP-treated groups, where the protein levels showed significant reduction (Figure 3F,G), while the mRNA levels remained relatively unchanged compared to the controls (Figure 3J,K). This observation suggests complex post-translational regulation mechanisms, potentially involving enhanced protein degradation via the ubiquitin–proteasome system in APAP-treated groups. The presence of iron-responsive elements (IREs) in *FTH1* and *FTL1* mRNAs, which can bind iron regulatory proteins (IRPs) under conditions of iron deficiency or oxidative stress, may contribute to translational repression. Additionally, APAP-induced oxidative stress might affect protein stability through oxidative modifications, leading to increased protein degradation. Immunohistochemical staining further corroborated these findings, showing an enhanced expression of FTH1, FTL1, and FPN1 in both 4-OI alone and APAP + 4-OI groups (Figure 3M). Collectively, these results demonstrate that 4-OI exhibits dual mechanisms in regulating iron homeostasis: it both reverses APAP-induced iron dysregulation and independently promotes iron storage and export, ultimately protecting against ferroptotic liver injury.

### 3.4. 4-OI Modulates Ferroptosis-Related Gene Expression and Exhibits Selective Protection Against APAP-Induced Liver Injury

**Figure 4:**
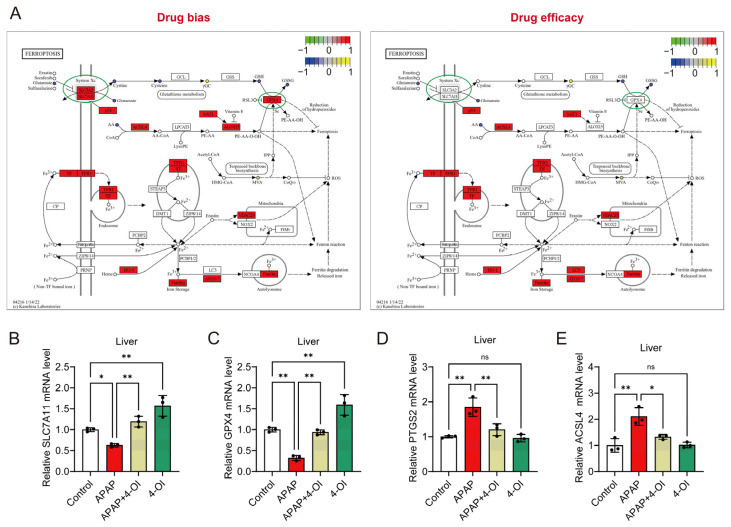
4-OI attenuates APAP-induced ferroptosis by modulating the expression of ferroptosis pathway genes. (**A**) Illustration of the ferroptosis pathway. (**B**–**E**) RT-qPCR detection of hepatic mRNA expression for *SLC7A11*, *GPX4*, *PTGS2*, and *ACSL4* in liver (*n* = 3). * *p* < 0.05, ** *p* < 0.01; ns: not significant.

A pathway enrichment analysis revealed distinct patterns between drug bias and efficacy markers in the ferroptosis pathway (Figure 4A). The bias-associated metabolites showed stronger enrichment in ferroptosis-related pathways compared to efficacy-associated metabolites, suggesting differential regulation of these processes. Notably, the key ferroptosis regulators SLC7A11 and GPX4 demonstrated more pronounced responses within the bias-associated network, while the efficacy-related pathways showed relatively modest changes in response to 4-OI pretreatment in APAP-challenged mice. To validate these pathway-specific effects, we examined the hepatic expression of ferroptosis-related genes. The mRNA analysis revealed two distinct response patterns. First, markers associated with drug bias, *SLC7A11* and *GPX4*, showed significant upregulation in APAP + 4-OI-treated mice compared to APAP treatment (Figure 4B,C). Second, the efficacy-related markers *PTGS2* and *ACSL4* exhibited decreased expression following 4-OI administration (Figure 4D,E). These divergent expression patterns suggest that 4-OI’s protective effects against APAP-induced liver injury operate through the selective modulation of ferroptosis pathways, primarily by enhancing the expression of anti-ferroptotic factors (*SLC7A11*, *GPX4*) while simultaneously suppressing pro-ferroptotic mediators (*PTGS2*, *ACSL4*).

### 3.5. Coordinated Upregulation of SLC7A11 and OTUB1 Mediates 4-OI’s Hepatoprotective Effects

**Figure 5:**
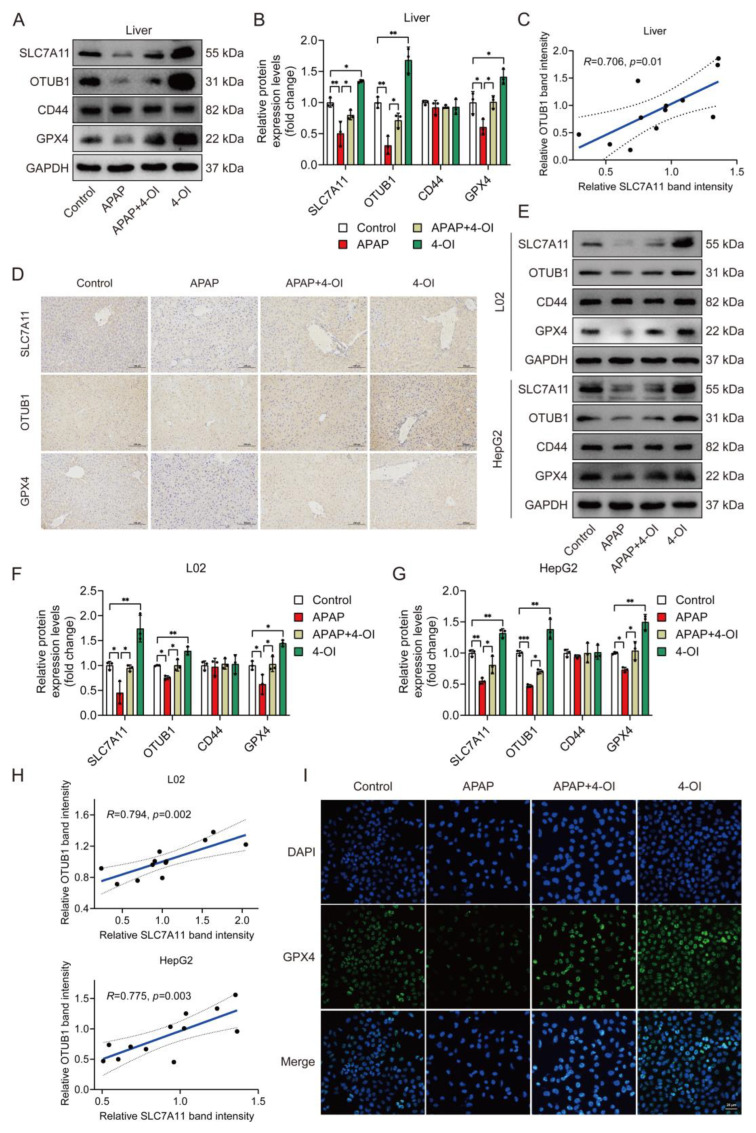
4-OI upregulates the anti-ferroptotic proteins SLC7A11, OTUB1, and GPX4 in the livers of APAP-treated mice. (**A**,**B**) Western blot analysis and quantification of protein levels of SLC7A11, OTUB1, CD44, and GPX4 in liver tissues (*n* = 3). (**C**) Pearson’s correlation test determined a positive correlation between SLC7A11 expression and OTUB1 expression in liver tissues. (**D**) Immunohistochemical staining of SLC7A11, OTUB1, and GPX4 protein expression (*n* = 3). Scale bar: 100 μm. (**E**–**G**) Western blot analysis and quantification of protein levels of SLC7A11, OTUB1, GPX4, and CD44 in L02 and HepG2 cells (*n* = 3). (**H**) A positive correlation between SLC7A11 expression and OTUB1 expression was determined using Pearson’s correlation test in L02 and HepG2 cells. (**I**) Immunofluorescence images showing GPX4 expression in L02 cells. Scale bar: 25 μm, *n* = 3. GAPDH was used as internal loading control. * *p* < 0.05, ** *p* < 0.01, *** *p* < 0.001.

Given that SLC7A11 protein stability is regulated by the deubiquitylase OTUB1 in a CD44-dependent manner, with CD44 serving as a critical mediator of the OTUB1-SLC7A11 interaction [15,16], we investigated the expression patterns of these regulatory proteins alongside SLC7A11 and GPX4. A Western blot analysis revealed that APAP treatment significantly decreased hepatic SLC7A11 protein levels compared to controls (Figure 5A,B). Importantly, 4-OI treatment not only reversed this APAP-induced decrease but enhanced SLC7A11 expression beyond baseline levels, suggesting a therapeutic mechanism extending beyond simple restoration. This enhancement pattern was mirrored by OTUB1, while the CD44 levels remained unchanged across the treatment groups. 4-OI also effectively prevented the APAP-induced reduction in GPX4 protein levels. A correlation analysis revealed a significant positive relationship between SLC7A11 and OTUB1 expression (Figure 5C), suggesting coordinated regulation. An immunohistochemical analysis of the liver sections confirmed these findings, showing enhanced expression of SLC7A11, OTUB1, and GPX4 in 4-OI-treated mice (Figure 5D). To validate these in vivo observations, we examined these effects in human hepatic cell lines. Both L02 and HepG2 cells demonstrated similar expression patterns following 4-OI treatment (Figure 5E–H), with significant correlations between SLC7A11 and OTUB1 expressions. Immunofluorescence analysis further confirmed increased GPX4 expression in L02 cells after 4-OI treatment (Figure 5I). These findings demonstrate that 4-OI’s protective effects involve a coordinated upregulation of SLC7A11 and OTUB1, maintaining elevated GPX4 levels in both in vivo and in vitro models of APAP-induced liver injury. The strong correlation between SLC7A11 and OTUB1 expression suggests a mechanistic link in their regulation by 4-OI.

### 3.6. 4-OI Stabilizes SLC7A11 Through OTUB1-Mediated Deubiquitination in APAP-Induced Liver Injury

**Figure 6:**
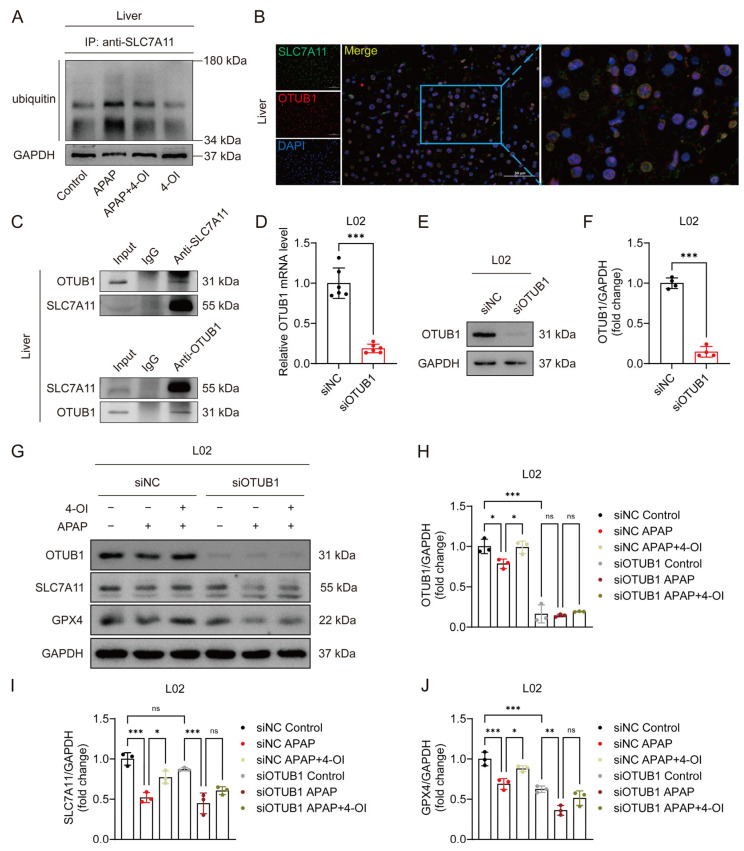
4OI restores SLC7A11 expression through OTUB1-mediated deubiquitination in APAP-induced liver injury. (**A**) CoIP assays determined the ubiquitination levels of SLC7A11 (*n* = 3). (**B**) Immunofluorescence analysis of SLC7A11 (green) and OTUB1 (red) expression and co-localization (yellow) in liver sections after 4-OI pretreatment in APAP-treated mice. Nuclei were stained with DAPI (blue). Scale bar: 50 μm, *n* = 3. (**C**) Western blot analysis of OTUB1/ SLC7A11 after Co-IP of anti-SLC7A11/anti-OTUB1 from liver tissue in APAP group treated with 4-OI (*n* = 3). (**D**) Relative mRNA level of *OTUB1* in L02 cells transfected with siRNA against OTUB1 (*n* = 6). (**E**,**F**) Western blot analysis and quantification of protein level of OTUB1 with or without siOTUB1 transfection (*n* = 4). (**G**–**J**) Western blot analysis and quantification of protein levels of OTUB1, SLC7A11, and GPX4 with or without siOTUB1 transfection (*n* = 3). GAPDH was used as internal loading control. * *p* < 0.05, ** *p* < 0.01, *** *p* < 0.001; ns: not significant.

OTUB1, a critical deubiquitinating enzyme, has been previously demonstrated to directly interact with and stabilize SLC7A11 through its deubiquitinase activity in multiple cellular contexts [16]. Given the established role of OTUB1 in SLC7A11 regulation, we investigated whether 4-OI’s protective effects against APAP-induced liver injury involve OTUB1-mediated SLC7A11 stabilization. Western blot analysis revealed that APAP treatment significantly enhanced SLC7A11 ubiquitination, leading to protein destabilization and reduced SLC7A11 levels in mouse liver tissues. Importantly, 4-OI administration effectively counteracted this effect by substantially decreasing APAP-induced SLC7A11 ubiquitination, thereby restoring its protein stability (Figure 6A). Immunofluorescence imaging demonstrated a strong colocalization of SLC7A11 and OTUB1 in liver sections from 4-OI-pretreated mice (Figure 6B), suggesting their spatial proximity. Co-IP experiments using both anti-SLC7A11 and anti-OTUB1 antibodies confirmed a robust physical interaction between these proteins in liver tissues following 4-OI pretreatment (Figure 6C). To establish the functional significance of OTUB1 in 4-OI-mediated hepatoprotection, we performed siRNA-mediated OTUB1 knockdown in L02 hepatocytes. OTUB1’s silencing efficiency was verified at both the mRNA and protein levels (Figure 6D–F). Notably, OTUB1 depletion reduced basal SLC7A11 and GPX4 expression levels (Figure 6G–J). More importantly, OTUB1 knockdown significantly attenuated 4-OI’s ability to restore SLC7A11 and GPX4 expression in APAP-treated cells (Figure 6G–J), indicating that OTUB1 is essential for 4-OI’s protective effects. These findings demonstrate that 4-OI exerts its hepatoprotective effects by enhancing OTUB1-mediated deubiquitination and stabilization of SLC7A11, thereby maintaining cellular antioxidant capacity against APAP-induced ferroptosis.

### 3.7. SLC7A11 Function Is Required for Optimal 4-OI-Mediated Enhancement of Cellular Glutathione Synthesis

**Figure 7:**
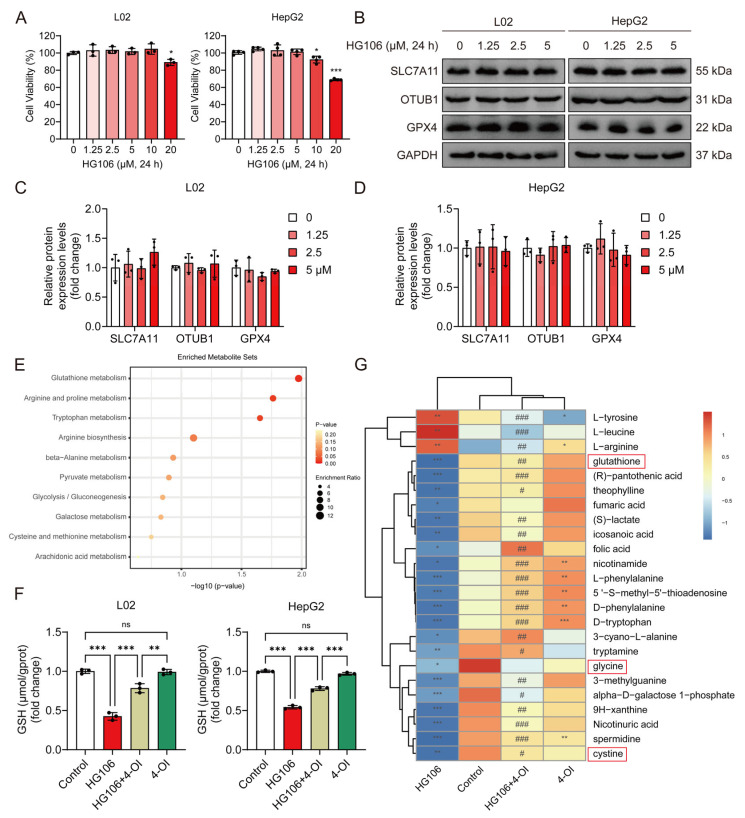
4-OI upregulates SLC7A11 to enhance cystine import and glutathione synthesis. (**A**) MTT assay evaluated the viability of L02 and HepG2 cells that were treated with different concentrations (0, 1.25, 2.5, 5, 10, and 20 μM) of HG106 for 24 h (*n* = 3). * *p* < 0.05, *** *p* < 0.001 compared with the untreated cells group. (**B**–**D**) Western blot analysis and quantification of protein levels of SLC7A11, OTUB1, and GPX4 in L02 and HepG2 cells (*n* = 3). GAPDH was used as internal loading control. (**E**) Metabolomic profiling of L02 cell lysates pretreated with 25 μM 4-OI for 12 h, followed by treatment with 5 μM HG106 for an additional 6 h. Pathway enrichment analysis identified top disrupted and recovered routes. (**F**) L02 and HepG2 cells were pretreated with 4-OI (25 μM) for 12 h, followed by HG106 (5 μM) exposure for 12 h. The intracellular GSH levels were measured using a GSH and GSSG assay kit (*n* = 3). ** *p* < 0.01, *** *p* < 0.001; ns: not significant. (**G**) Heatmap of differentially abundant metabolites central to glutathione biosynthesis (*n* = 7). Metabolites involved in glutathione metabolism are highlighted in red boxes. Metabolite levels are represented by blue (low) and red (high). Statistical significance (Benjamini–Hochberg-adjusted) was determined via two-tailed Student’s *t*-test or Mann–Whitney test, as appropriate. * *p* < 0.05, ** *p* < 0.01, *** *p* < 0.001 compared with the control group; # *p* < 0.05, ## *p* < 0.01, ### *p* < 0.001 compared with the HG106 group.

To determine whether SLC7A11 is essential for 4-OI’s protective effects, we employed HG106, a selective SLC7A11 inhibitor. We first established safe working concentrations by conducting cytotoxicity studies in L02 and HepG2 cells. Cell viability assays revealed that concentrations up to 5 μM HG106 maintained cell viability above 90% after 24 h treatment, while higher concentrations (10–20 μM) showed significant cytotoxicity (Figure 7A). Based on these findings, we selected 0–5 μM as the optimal concentration range for the subsequent experiments. A Western blot analysis demonstrated that HG106 treatment within this concentration range did not affect the protein expression of SLC7A11, OTUB1, or GPX4 (Figure 7B–D), indicating that HG106 inhibits SLC7A11 function rather than expression. Given the intimate connection between ferroptosis and cellular redox state, we performed untargeted metabolomic profiling to comprehensively assess the impact of HG106 and 4-OI on cellular metabolism. A pathway enrichment analysis revealed that glutathione metabolism was the most significantly affected pathway, followed by several amino acid metabolism pathways crucial for redox homeostasis (Figure 7E). Notably, HG106 treatment significantly decreased the intracellular GSH levels in both cell lines (Figure 7F). A detailed metabolite analysis showed that HG106 treatment markedly decreased the levels of cystine and its downstream metabolites, including glycine and glutathione, confirming effective SLC7A11 inhibition (Figure 7G). While 4-OI treatment alone significantly elevated GSH levels through Nrf2 activation, the combination of HG106 and 4-OI showed substantially lower GSH levels compared to 4-OI alone, indicating that SLC7A11 inhibition partially blocks 4-OI’s ability to enhance GSH synthesis. This was further supported by reduced levels of glycine and other GSH synthesis precursors in the HG106 + 4-OI group compared to 4-OI alone, demonstrating that functional SLC7A11 is required for optimal 4-OI-mediated GSH synthesis.

### 3.8. Nrf2 Partially Mediates 4-OI’s Anti-Ferroptotic Effects in APAP-Induced Hepatotoxicity

**Figure 8:**
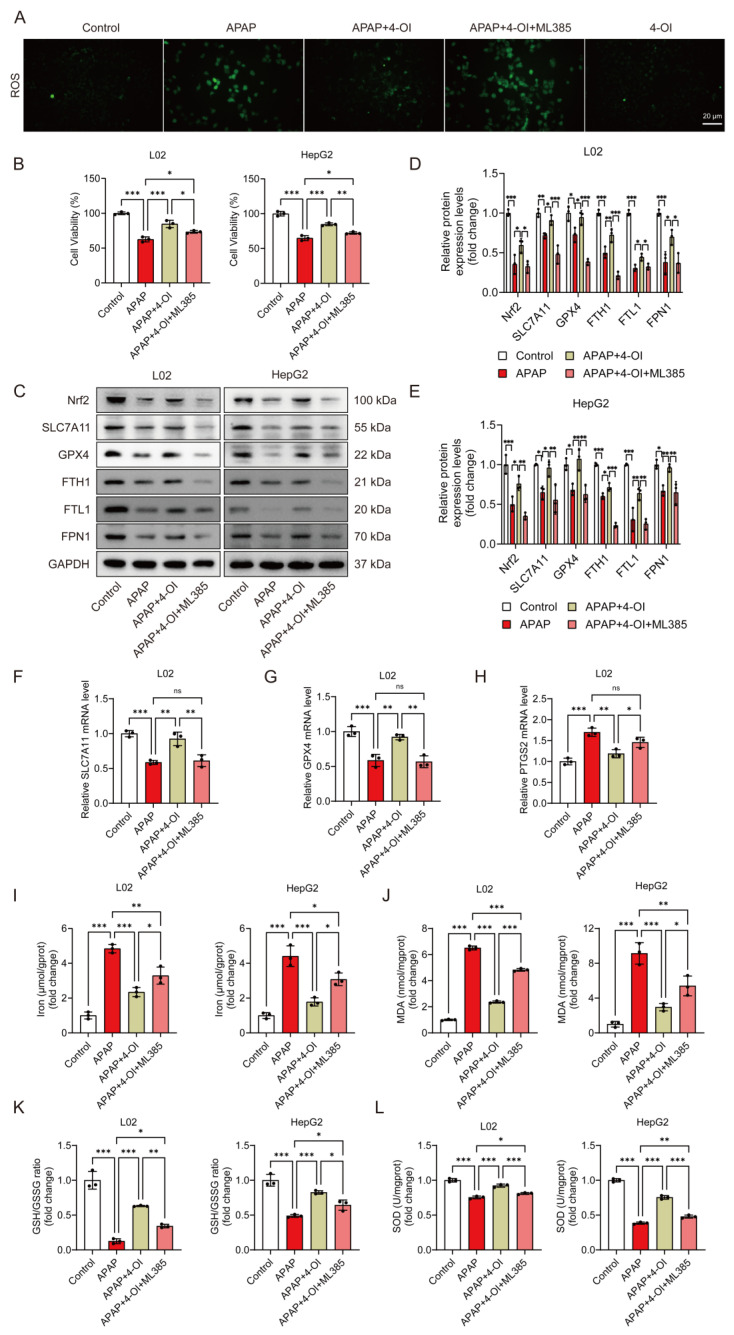
Nrf2 inhibition partially abolished the protective effects of 4-OI against APAP-induced ferroptosis in hepatic cells. L02 and HepG2 cells were pretreated with 25 μM 4-OI for 12 h, followed by APAP exposure for 24 h. The specific Nrf2 inhibitor ML385 (5 μM) was added 2 h before 4-OI treatment. (**A**) ROS levels in L02 cells. Scale bar: 20 μm, *n* = 3. (**B**) MTT assay evaluated the viability of L02 and HepG2 cells (*n* = 3). (**C**–**E**) Western blot analysis and quantification of protein levels of Nrf2, SLC7A11, GPX4, FTH1, FTL1, and FPN1 (*n* = 3). (**F**–**H**) Relative mRNA levels of *SLC7A11*, *GPX4* and *PTGS2* (*n* = 3). (**I**) Iron content (*n* = 3). (**J**–**L**) MDA levels, GSH/GSSG ratio, and SOD activities (*n* = 3). GAPDH was used as internal loading control. * *p* < 0.05, ** *p* < 0.01, *** *p* < 0.001; ns: not significant.

We previously demonstrated that 4-OI induced Nrf2 activation in APAP-treated mice [14]. To investigate the contribution of Nrf2 activation to 4-OI’s anti-ferroptotic effects, we employed ML385, a specific Nrf2 inhibitor. ROS fluorescence imaging confirmed ML385’s effectiveness in blocking Nrf2 transcriptional activity, as evidenced by significantly higher ROS levels in the APAP + 4-OI + ML385 group compared to both the APAP and APAP + 4-OI groups (Figure 8A). Cell viability assays revealed that ML385 pretreatment partially reversed 4-OI’s protective effects against APAP-induced injury in both L02 and HepG2 cells (Figure 8B). At the molecular level, ML385 not only prevented 4-OI-mediated upregulation of Nrf2 but also suppressed the expression of its downstream targets, including SLC7A11, GPX4, and the iron metabolism regulators FPN1, FTH1, and FTL1 (Figure 8C–E). Intriguingly, despite the protein levels of SLC7A11, GPX4, FPN1, and FTH1 in the APAP + 4-OI + ML385 group falling below those in the APAP group, cell viability remained higher than in the APAP-treated cells. This discrepancy suggests that the absolute expression levels of these proteins may not be the determining factor in ferroptotic cell death. ML385 also reversed 4-OI’s effects on *SLC7A11* and *GPX4* mRNA expression while increasing the ferroptosis marker *PTGS2* (Figure 8F–H). Importantly, an analysis of ferroptosis-related parameters revealed that ML385 significantly impacted cellular redox status and iron homeostasis. While 4-OI pretreatment effectively reduced the iron content and MDA levels while increasing the GSH/GSSG ratio and SOD activity in APAP-treated cells, ML385 addition partially reversed these protective effects (Figure 8I–L). Notably, the levels of these markers in the APAP + 4-OI + ML385 group remained intermediate between the APAP and APAP + 4-OI groups, suggesting that Nrf2 inhibition could not completely abolish 4-OI’s protective effects. This partial reversal indicates that, while Nrf2 activation contributes to 4-OI’s antioxidant effects, 4-OI likely employs additional mechanisms upstream of ferroptosis to achieve hepatoprotection.

## 4. Discussion

In this study, we have uncovered novel mechanistic insights into how 4-OI protects against APAP-induced ferroptosis through comprehensive metabolomic profiling and molecular analyses. Our findings reveal that 4-OI operates through multiple coordinated pathways to prevent ferroptotic liver injury, with both direct and indirect effects on key regulatory molecules.

The metabolomic analysis revealed two distinct response patterns to 4-OI treatment: drug bias-related and efficacy-related metabolic changes. This dual action is particularly evident in the regulation of iron homeostasis, where 4-OI both reverses APAP-induced perturbations and independently modulates iron metabolism under physiological conditions. Dysregulated iron metabolism is a key contributor to ferroptosis [17], as iron overload can exacerbate lipid peroxidation and mitochondrial damage by promoting the Fenton reaction [18]. The maintenance of cellular iron balance involves precisely coordinated mechanisms governing iron import, export, and storage, primarily mediated by TFR1, FPN1, and ferritin complexes [19]. TFR1, a cell surface glycoprotein, facilitates the endocytosis of transferrin-bound iron and its subsequent release into the cytoplasmic compartment. The released cytoplasmic iron is then safely incorporated into ferritin-a multimeric protein complex composed of 24 subunits that include both heavy (FTH1) and light (FTL1) chain components, which function as the primary intracellular iron storage system. The ferrous iron (Fe^2+^) internalized by hepatocytes is partly utilized by the cells and partly exported into the bloodstream via FPN1. In this study, the coordinated upregulation of iron storage proteins (FTH1, FTL1) and the iron exporter FPN1, alongside decreased transferrin and hepcidin levels, suggests that 4-OI promotes both iron sequestration and export. This two-pronged approach effectively reduces the pool of free iron available for Fenton reactions, thereby limiting lipid peroxidation and subsequent ferroptotic damage [20]. Our data reveal an interesting pattern where 4-OI treatment affects the iron distribution between the tissue and serum compartments. Specifically, serum iron analysis demonstrates that 4-OI treatment helps maintain normal serum iron levels in both erastin and APAP-induced liver injury models, preventing the significant decrease observed in untreated conditions. This maintenance of serum iron levels suggests that 4-OI influences the hepcidin–FPN1 regulatory axis. The mechanism appears to involve 4-OI’s effect on hepcidin expression, which subsequently impacts FPN1 functionality. When hepcidin levels decrease, there is reduced binding to FPN1, resulting in less internalization and degradation of this crucial iron exporter [21]. This enhanced FPN1 stability facilitates increased iron export from cells into the bloodstream, consistent with the serum iron data. Supporting this interpretation, liver iron content measurements demonstrate reduced tissue iron accumulation in 4-OI-treated groups compared to erastin or APAP treatment alone. The reciprocal relationship between tissue and serum iron levels clearly illustrates the dynamic regulation of iron homeostasis through the hepcidin–FPN1 pathway under 4-OI treatment. These findings indicate that 4-OI’s protective effects against liver injury may partially operate through a modulation of iron homeostasis, maintaining appropriate iron distribution between the cellular and systemic compartments. This mechanism appears to be an important component of 4-OI’s therapeutic potential in liver injury conditions.

SLC7A11 and GPX4 serve as key regulators in ferroptosis [22,23,24]. SLC7A11 plays a pivotal role in ferroptosis by regulating cystine uptake and glutathione synthesis [25,26], while GPX4 functions as an antioxidant enzyme that inhibits ferroptosis by reducing lipid peroxides using GSH as a cofactor [27,28,29,30,31]. Additionally, PTGS2 (also known as cyclooxygenase-2) is elevated in ferroptotic cells [29], while ACSL4, functioning as a positive regulator of ferroptosis, promotes lipid ROS accumulation and subsequent ferroptotic cell death [32]. Interestingly, the differential expression patterns of ferroptosis-related genes (SLC7A11 and GPX4 versus PTGS2 and ACSL4) indicate that 4-OI not only enhances the protective mechanisms but also actively suppresses pro-ferroptotic pathways. This bilateral regulation represents a more comprehensive approach to ferroptosis prevention compared to strategies that target only protective or damaging pathways in isolation.

A key finding of our study is the identification of the OTUB1-SLC7A11 axis as a critical mediator of 4-OI’s protective effects. While previous studies have established SLC7A11’s role in ferroptosis prevention through cystine import and GSH synthesis, our results reveal a novel regulatory mechanism involving OTUB1-mediated deubiquitination. The strong correlation between SLC7A11 and OTUB1 expression, coupled with their physical interaction following 4-OI pretreatment, suggests that 4-OI enhances SLC7A11 stability through post-translational modification rather than merely increasing its expression. This mechanism is particularly significant as it provides a rapid response system to maintain cellular antioxidant capacity under stress conditions. The metabolomic data further support this mechanism, showing that 4-OI pretreatment restores glutathione-related metabolites and normalizes inflammatory mediators. The observation that SLC7A11 inhibition by HG106 partially blocks 4-OI’s ability to enhance GSH synthesis demonstrates the essential role of SLC7A11 function in 4-OI’s protective effects. This finding bridges the gap between protein stabilization and metabolic outcomes, providing a mechanistic link between 4-OI’s molecular targets and its physiological effects.

Nrf2 is a transcriptional factor that regulates the antioxidant response element (ARE) pathway to coordinate cellular antioxidant defenses [33,34]. Under normal conditions, Keap 1 tightly regulates Nrf2 levels by mediating its ubiquitination and proteasomal degradation, maintaining low protein expression [35]. Upon stimulation, the Nrf2-dependent defense system is activated via the dissociation of Nrf2 from Keap 1, enabling its nuclear translocation and activation of target genes [36]. Notably, Nrf2 not only serves as a pivotal regulator of cellular antioxidant defenses but also exerts an inhibitory effect on ferroptosis by modulating the expression of genes involved in iron metabolism, such as ferritin and FPN1 [37]. The involvement of Nrf2 adds another layer of complexity to 4-OI’s mechanism of action. Our results with ML385 demonstrate that Nrf2 activation is necessary but not sufficient for 4-OI’s full protective effects. The partial reversal of 4-OI’s benefits by Nrf2 inhibition, particularly in terms of antioxidant capacity and iron homeostasis, suggests that 4-OI operates through both Nrf2-dependent and independent pathways. Our study primarily reveals the novel OTUB1-SLC7A11 regulatory axis as a key mechanism underlying 4-OI-mediated protection against oxidative stress and ferroptosis. However, it is important to note that 4-OI exhibits multiple mechanisms of action beyond Nrf2 activation. Among these Nrf2-independent pathways, 4-OI has been shown to inhibit GAPDH through the alkylation of cysteine residue 22, leading to a decreased glycolytic flux and an increased oxygen consumption rate [13]. This metabolic modulation also contributes to its anti-inflammatory and cytoprotective effects. The multi-targeted nature of 4-OI’s action may explain its robust therapeutic efficacy, as cellular protection can be maintained even when individual pathways are compromised.

The coordinated regulation of multiple pathways, including iron homeostasis, antioxidant defense, and protein stability, suggests that 4-OI acts as a master regulator of cellular stress responses. The integration of these pathways provides redundancy and robustness to the protective mechanism, potentially explaining why 4-OI maintains some protective effects even when individual pathways are blocked.

## 5. Conclusions

In conclusion, our study reveals that 4-OI protects against APAP-induced hepatotoxicity through a sophisticated network of interacting pathways, centered on the OTUB1-SLC7A11 axis and modulated by Nrf2 signaling. This comprehensive understanding of 4-OI’s mechanism of action not only provides new insights into ferroptosis regulation but also suggests potential therapeutic strategies for other conditions involving oxidative stress and cell death. Future studies might explore whether similar mechanisms operate in other tissue types and disease contexts where ferroptosis plays a pathogenic role.

## Data Availability

The authors declare that any supporting data or material associated with this original research is available from the corresponding authors under reasonable request.

## Supplementary Materials

The following supporting information can be downloaded at https://www.mdpi.com/article/10.3390/antiox14060698/s1: Figure S1: 4-OI reduces APAP-induced pro-inflammatory cytokine expression and restores redox balance in liver tissues; Figure S2: 4-OI protects against APAP- and erastin-induced liver injury in vivo; Table S1: The primers used in this study.

## Abbreviations

The following abbreviations are used in this manuscript:

4-OI	4-octyl itaconate
ALT	alanine aminotransferase
ANOVA	one-way analysis of variance
APAP	acetaminophen
AST	aspartate aminotransferase
BSA	bovine serum albumin
CE	collision energy
Co-IP	co-immunoprecipitation
Cy3	cyanine 3
DAB	3,3′-diaminobenzidine
ECL	enhanced chemiluminescence
ESI	electrospray ionization
FBS	fetal bovine serum
FITC	fluorescein isothiocyanate
FPN1	ferroportin 1
GSH	glutathione
HMDB	Human Metabolome Database
HRP	horseradish peroxidase
IDA	information-dependent acquisition
IHC	immunohistochemistry
IRG1	immunoresponsive gene 1
MSEA	metabolic set enrichment analysis
NAPQI	N-acetyl-p-benzoquinone imine
NC	nitrocellulose
NEM	N-ethylmaleimide
PLS-DA	partial least squares discriminant analysis
RT-qPCR	real-time quantitative PCR
SD	standard deviation
TCA	tricarboxylic acid
TFR1	transferrin receptor 1
