# Supplementary material for: OTUB1-SLC7A11 Axis Mediates 4-Octyl Itaconate Protection Against Acetaminophen-Induced Ferroptotic Liver Injury
Source: Antioxidants (Basel). 2025 Jun 9;14(6):698. doi: 10.3390/antiox14060698 (PMC12189733; doi:10.3390/antiox14060698)
Supplement: Supplementary file 1 [file antioxidants-14-00698-s001.zip › antioxidants-3622848-supplementary.pdf]

antioxidants

Supplementary Material: OTUB1-SLC7A11 axis mediates 4-octyl itaconate protection against acetaminophen-induced ferroptotic liver injury

Ziyun Hu †, Yuxin Li †, Di Xu, Huihui Meng, Wenya Liu, Qian Xu, Benxing Yao and Junsong Wang *

Center for Molecular Metabolism, Nanjing University of Science and Technology, 200 Xiao Ling Wei Street, Nanjing 210094, China; hzy@njust.edu.cn (Z.H.); liyuxin@njust.edu.cn (Y.L.); xudi@njust.edu.cn (D.X.); menghuihui2014@gmail.com (H.M.); wenyaliu2015@163.com (W.L.); zhiqidangran@gmail.com (Q.X.); yaobenxing@njust.edu.cn (B.Y.)
* Correspondence: wangjunsong@njust.edu.cn; Tel./Fax: +86-139-1397-6393
† These authors contributed equally to this work.

Supplemental Tables

Table S1. The primers used in this study.

Gene name	Gene accession	Forward (5′-3′)	Reverse (5′-3′)
Mouse-FPN1	NM_016917.2	CCATAGTCTCTGTCAGCCTGCT	CTTGCAGCAACTGTGTCACCGT
Mouse-FTH1	NM_010239.2	GCCGAGAAACTGATGAAGCTGC	GCACACTCCATTGCATTCAGCC
Mouse-FTL1	NM_010240.2	CCTCGAGTTTCAGAACGATCGC	CCTGATTCAGGTTCTTCTCCATG
Mouse-SLC7A11	NM_011990.2	CTTTGTTGCCCTCTCCTGCTTC	CAGAGGAGTGTGCTTGTGGACA
Mouse-GPX4	NM_0010377 41.4	CCTCTGCTGCAAGAGCCTCCC	CTTATCCAGGCAGACCATGTGC
Mouse-ACSL4	NM_207625.2	CCTTTGGCTCATGTGCTGGAAC	GCCATAAGTGTGGGTTTCAGTAC
Mouse-PTGS2	NM_011198.5	GCGACATACTCAAGCAGGAGCA	AGTGGTAACCGCTCAGGTGTTG
Mouse-TNF-α	NM_013693.3	GATCGGTCCCCAAAGGGATG	TTTGCTACGACGTGGGCTAC
Mouse-IL-6	NM_031168.2	CCCCAATTTCCAATGCTCTCC	AGGTTTGCCGAGTAGATCTCAA
Mouse-IL-1β	NM_008361.4	ATGCCACCTTTTGACAGTGATG	TGTGCTGCTGCGAGATTTGA
Mouse-GAPDH	NM_0012897 26.2	CATCACTGCCACCCAGAAGACTG	ATGCCAGTGAGCTTCCCGTTCAG
Human-SLC7A11	NM_014331.4	TGGTCAGAAAGCCTGTTGTGT	TGCTCCAATGATGGTGCCAA
Human-GPX4	NM_0010398 47.3	AGGAGCCAGGGAGTAACGAA	CGGTGTCCAAACTTGGTGAAG
Human-PTGS2	NM_000963.4	TGCGCCTTTTCAAGGATGGA	CCCCACAGCAAACCGTAGAT
Human-GAPDH	NM_002046.7	ACAACTTTGGTATCGTGGAAGG	GCCATCACGCCACAGTTTC

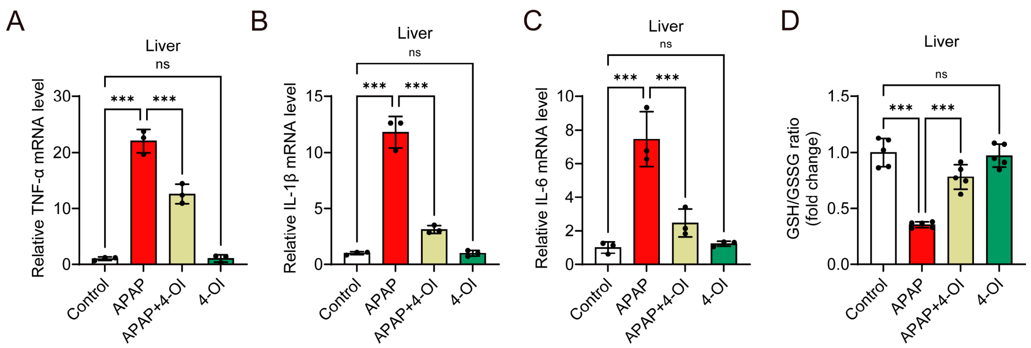

Figure S1. 4-OI reduces APAP-induced pro-inflammatory cytokine expression and restores redox balance in liver tissues. **(A-C)** A RT-qPCR assay determined the mRNA expression of *TNF-α*, *IL-1β*, and *IL-6* (n = 3). **(D)** GSH/GSSG ratio (n = 5). ***p < 0.001; ns: not significant.

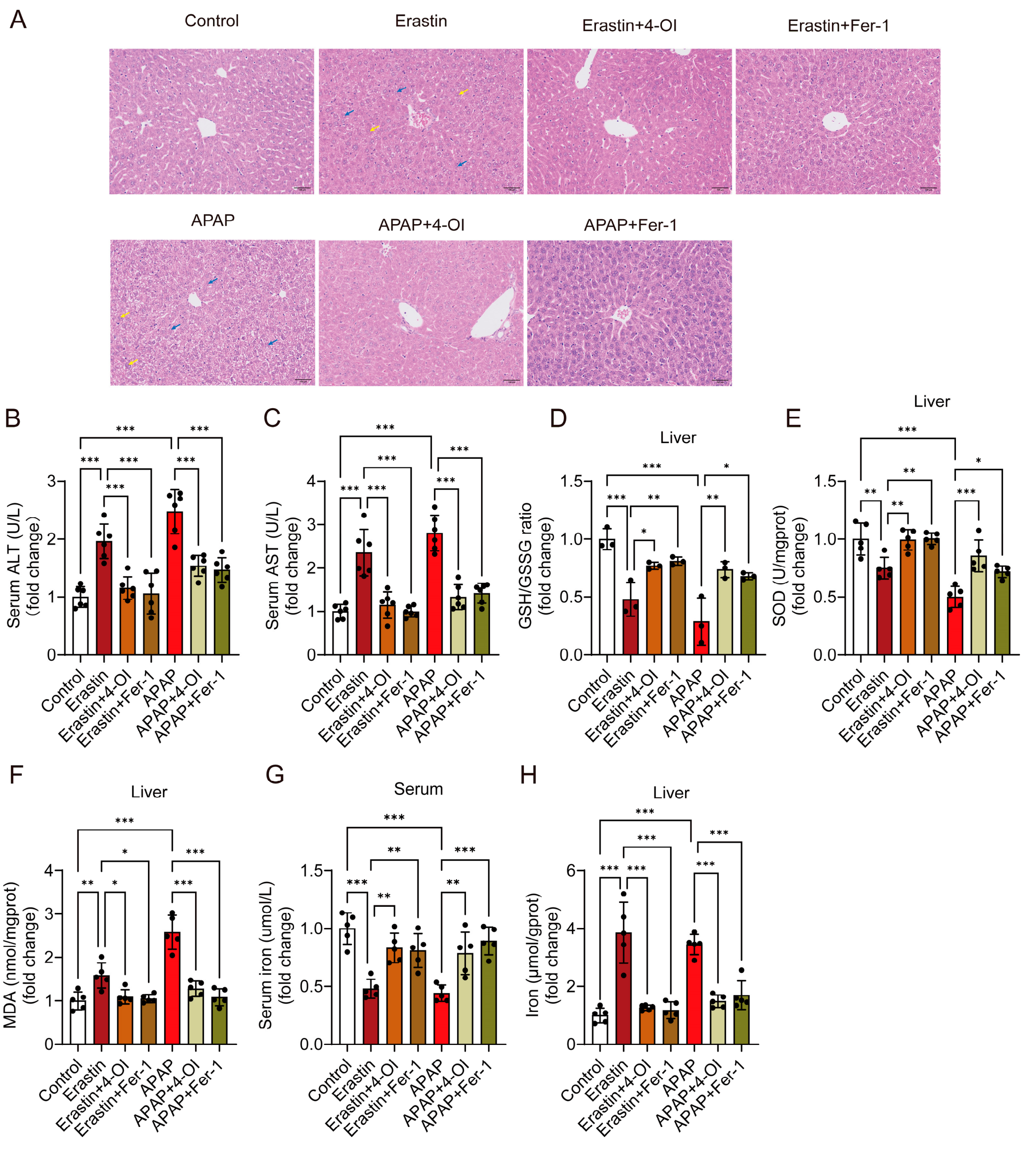

Figure S2. 4-OI protects against erastin- and APAP-induced liver injury in vivo. **(A)** H&E staining evaluated histopathological changes in liver sections. Blue arrows indicate necrotic cells showing classical morphological features including nuclear pyknosis, cytoplasmic condensation, and loss of membrane integrity; Yellow arrows highlight inflammatory infiltrating cells. (B, C) Serum levels of ALT and AST (n = 6). **(D)** Ratio of GSH to GSSG in liver tissues (n = 3). **(E)** SOD activity in liver tissues (n = 5). **(F)** MDA levels in liver tissues (n = 5). (G) Serum iron levels (n = 5). **(H)** Iron content in liver tissues (n = 5). *$p < 0.05$, **$p < 0.01$, ***$p < 0.001$.
